# Avoidant/Restrictive Food Intake Disorder in Celiac Disease

**DOI:** 10.3390/nu17203197

**Published:** 2025-10-11

**Authors:** Ridhima Kaul, Claire Jansson-Knodell, Madison L. Simons, Kendra Weekley, David Gardinier, Alberto Rubio-Tapia

**Affiliations:** 1 Department of Internal Medicine, Cleveland Clinic Foundation, Cleveland, OH 44195, USA; kaulr2@ccf.org; 2 Celiac Disease Program, Department of Gastroenterology, Hepatology and Nutrition, Digestive Disease Institute, Cleveland Clinic, Cleveland, OH 44195, USA

**Keywords:** celiac disease, ARFID, gluten free diet, disordered eating

## Abstract

Celiac disease (CeD) is an autoimmune disorder where adherence to a lifelong gluten-free diet (GFD) is the only available treatment. While this approach is rather effective, some patients experience ongoing symptoms, and this factor, along with the rigidity of the GFD, may predispose some to disordered eating behaviors, including Avoidant/Restrictive Food Intake Disorder (ARFID). ARFID is characterized by persistent food avoidance that is not driven by body image concerns, resulting in nutritional, psychological, and social impairment. This scoping literature review explores the emerging intersection between ARFID and CeD, examining prevalence, pathophysiology, clinical features, complications, and management strategies. Recent studies report that 14–57% of individuals with CeD may meet the criteria for ARFID, depending on the population and screening tools used. Factors contributing to ARFID in CeD may include ongoing gastrointestinal symptoms, anxiety over gluten exposure, negative conditioned responses to food, social challenges related to GFD adherence, and psychiatric co-morbidities. ARFID in CeD is associated with worsened nutritional deficiencies, anxiety, depression, and impaired social functioning, making the diagnosis of ARFID challenging due to symptom overlap with CeD and other psychiatric conditions. Management requires a multidisciplinary approach, including medical, nutritional, and psychological interventions. Routine screening, early intervention, and integrated care models may improve outcomes and quality of life.

## 1. Introduction

Celiac disease (CeD) is an immune-mediated disorder triggered by a response to gluten present in wheat, rye, and barley. The prevalence of CeD was 0.71% in the United States in 2009–2010 [1], with an estimated 1.85:1 female-to-male predominance [2,3]. CeD presents with a broad range of clinical manifestations, often resembling a multisystemic disorder rather than a localized intestinal condition [2]. Exposure to gluten leads to inflammation in the small bowel and damages the surface area for nutrient absorption. The only effective treatment for CeD involves strict adherence to a gluten-free diet (GFD) and lifelong medical monitoring. A GFD promotes clinical, serological, and histologic improvement [2,4]. However, this diet is restrictive and requires daily diligence. Patients must consider if their food contains gluten each time they eat; it can be difficult to maintain, socially isolating, and mentally taxing.

Avoidant/Restrictive Food Intake Disorder (ARFID) is an eating disorder marked by persistent failure to meet one’s nutritional needs, often due to either a lack of interest in eating, sensory aversions to food, or concerns about the aversive consequences of eating [5,6,7,8]. Unlike anorexia nervosa or bulimia, ARFID is not driven by body image concerns, but rather is a form of food avoidance caused by heightened anxiety around eating. While the onset of ARFID can occur during childhood, where it is encountered by pediatric gastroenterologists and dietitians, this scoping literature review will primarily focus on adults. Patients with CeD may be predisposed to ARFID development if appropriate dietary caution to maintain a GFD evolves into dysfunctional beliefs or neophobia of food. A fear of the negative consequences of foods or food neophobias may not just lead to a narrowed diet, but potentially to ARFID. The overlap between the chronic gastrointestinal malabsorption in CeD and inadequate nutritional intake secondary to ARFID can lead to chronic nutrient deficiencies [5,7,8]. The management of ARFID in CeD requires a holistic, multi-disciplinary team approach, involving gastroenterologists, dietitians, and psychologists, to manage both the medical and psychosocial complications of the disease [6]. In this scoping literature review, we will cover the formal definition of ARFID, its epidemiology, pathophysiology, clinical features, complications, and management.

## 2. Review Methodology

A scoping literature review was performed on the concept of ARFID in the celiac disease population. The purpose of this was to describe the prevalence, etiology, clinical presentation, medical complications, and strategies for management. The goal was to provide a practical summary of this emerging intersection to increase awareness and aid clinicians in patient care. MEDLINE and Google Scholar were searched using the terms “ARFID” and “celiac disease”, with full-text articles included. Studies were excluded that were performed on populations other than celiac disease patients. Relevant data was extracted from the individual articles. Given the paucity of the literature in this area, the findings are reported in each relevant section, as opposed to in aggregate. It should be acknowledged that, due to scant original studies (often single-center and small in size) on this subject, the findings may not be generalizable to the broader celiac disease population. We aimed for a practical synthesis of the available research on this clinically relevant and under-explored topic.

## 3. Diagnosis

ARFID is a relatively new diagnosis, first introduced in the *Diagnostic and Statistical Manual of Mental Disorders* (*DSM-5*) in 2013 (Table 1) [9]. The key diagnostic feature of ARFID is the restriction or avoidance of food resulting in weight loss, nutrient deficiencies, or psychosocial interference. The symptoms of food avoidance should not be explained by lack of food, cultural or religious practices, or another concurrent medical disorder, nor should they be better explained by another mental disorder. Furthermore, the patient must not express body image concerns (related to weight, shape, or size), as seen in disorders like Anorexia Nervosa or Bulimia Nervosa.

ARFID can be trigged by other medical conditions such as food allergies or intolerances, especially conditions that manifest with restrictive eating or gastrointestinal symptoms (nausea, vomiting, diarrhea, abdominal pain, dysphagia). When ARFID occurs secondary to other medical conditions (such as in CeD), diagnosis requires that the disturbance in food intake is disproportionate to the medical condition. For example, in patients with CeD, celiac would be considered to be well controlled when there are no symptoms reported, normal serology, and biopsy showing intestinal healing, but the patient continues to restrict their diet beyond gluten-free foods. There is a difficulty in determining what is “disproportionate” to medical necessity, making this a potential area for further research and the development of new guidelines to provide physicians with specific and standardized diagnostic criteria for ARFID in the context of CeD.

## 4. Epidemiology of ARFID in Celiac Disease

The estimated prevalence of ARFID varies across different patient populations, but it is estimated that the prevalence of ARFID in the global adult population is between 0.3% and 3.1% [10]. Data on the incidence of ARFID in CeD remains scarce due to limited research specifically addressing this potential comorbid condition [10]. A recent retrospective patient-reported survey study based in Nashville, Tennessee, USA, of 137 patients (107 females, 30 males) with CeD seen in clinic over a 2-year period reported a prevalence of suspected ARFID in CeD of 57%. In total 21.9% of the study population had “clinical” ARFID, where they reported ≥1 ARFID symptoms (weight loss, nutritional deficiency, need for nutritional supplements, impaired daily life) on the ARFID checklist present “to a significant degree” and attributed their symptom to an ARFID cause (limited interest, picky eating, fear of negative consequence). The others had “sub-clinical” ARFID if ARFID symptoms were reported “to some degree.” This study may not be entirely generalizable to the CeD population, as it was single-center, based on patient survey responses as opposed to a clinical assessment, and women were over-represented.

A recent, similar cross-sectional study by Fink Et Al. used the NIAS (Nine-Item ARFID Screen) as a screen for ARFID in 76 adult patients with CeD. This screen tool utilizes questions of three different domains: sensory sensitivity (e.g., “I avoid foods with certain textures”), fear of aversive consequences (e.g., “I am afraid I might choke/vomit when I eat”), and lack of interest in eating (e.g., “I am rarely hungry or interested in eating”) to help identify symptoms consistent with ARFID [11]. They found that 37 (49%) of the patients had a result that indicated possible ARFID. This study featured self-reported measures at a tertiary care clinic for adults with various gastrointestinal conditions such as CeD, achalasia, eosinophilic esophagitis, and inflammatory bowel disease in Chicago, Illinois, USA. More than half of the total 289-patient sample screened positive for ARFID, highlighting that ARFID assessments may benefit from being individualized for those with complex gastrointestinal diseases. Authors emphasized the limitations of the NIAS in accurately distinguishing between pathological restriction and appropriate dietary caution in chronic GI conditions. In their study, they also found that patients who reported more ARFID symptoms had higher rates of anxiety, depression, and a lower quality of life. Interestingly, patients following a physician-prescribed diet or those who consulted with a dietitian exhibited higher total ARFID scores, possibly reflecting more severe underlying food-related anxiety or symptom burden [8].

Another study explored food neophobia in patients on a GFD with or without CeD in Poland. Food neophobia scale scores were significantly higher in those with CeD compared to those on a GFD of their own volition [12]. In a study of adults with CeD where ARFID status was unknown, 58% reported avoiding additional foods without diagnosed allergies or intolerances [4]. Fear and avoidance of foods beyond those containing gluten has been shown in those with CeD.

## 5. Etiology

The occurrence rate of ARFID in CeD noted above may seem high, and is perhaps overestimated by scales and screening tools developed on those without chronic gastrointestinal conditions. Whether this rate is inflated or not, certain aspects of CeD and its treatment could make patients susceptible to developing ARFID. Prior research suggests that eating disorders are, in general, more common in patients with CeD than in individuals without CeD [13,14,15,16,17]. This section will examine a hypothesis on how disproportionately restrictive eating might come to be in CeD patients.

The only effective treatment for CeD is the gluten-free diet (GFD); once initiated, the immune response is suppressed and the intestine begins to heal leading to resolution of clinical symptoms, normalization of serology, and reversal of histologic abnormalities. However, adhering to a strict GFD requires persistent vigilance from the patient, which could lead to a decreased quality of life and increased treatment burden. One survey study found that patients with CeD reported a high perceived burden of treatment, comparable to participants with end-stage renal disease and higher than those with gastroesophageal reflux disease or hypertension [18]. It is a challenging chronic condition to manage, requiring consideration with each meal or snack.

Part of managing CeD is restricting intake of dietary gluten entirely in order to treat it; theoretically, there is concern that the restrictive diet pattern needed for CeD treatment might trigger subtle maladaptive eating behaviors and an unhealthy relationship with food. If appropriate avoidance of gluten spills over into a generalized avoidance or restriction of food, this may be an indication of ARFID [18].

While food avoidance in ARFID may be secondary to several causes, such as the sensory characteristics of food or a lack of interest in eating, it is proposed that, for some patients, the food aversion in CeD is likely secondary to a conditioned negative response. The resolution of gastrointestinal symptoms after strictly adhering to a GFD and finally feeling well may increase anxiety and caution around diet plans and food preparation. Patients may be overly concerned and preoccupied with food labels, ingredients, and meal preparation due to concerns of feeling unwell if they experience accidental exposure to gluten. This may even manifest as an avoidance of social events: in one study, 94% of individuals diagnosed with CeD started bringing their own food to social gatherings, 81% opted out of dining out, and 38% avoided traveling [19]. Prior to the diagnosis of CeD, individuals may attempt to restrict their diets to identify the possible triggers of their symptoms. They may identify several food groups that trigger gastrointestinal symptoms, such as nausea, vomiting, bloating, gas, diarrhea, and abdominal pain or fullness. This behavior may motivate individuals with CeD to strictly limit their diet further than a GFD, even after they are diagnosed [4].

## 6. Potential Physician and Dietitian Role in Hypervigilance

CeD is unique from other gastrointestinal conditions in that the only available intervention is strict dietary avoidance. As such, physicians and dietitians who treat CeD encourage, and even expect, rigidity around gluten consumption. In CeD, 50 mg of gluten per day, the equivalent of a dime-sized amount of bread or crumbs, has been shown to produce significant damage to the small intestinal architecture of treated CeD patients [20]. Strict dietary limitations are necessary for treatment.

In other gastrointestinal conditions, such as irritable bowel syndrome (IBS), exposure to dietary irritants may cause severe discomfort, but patients can be reassured that these symptoms are not associated with harm to the body. However, a physician, dietitian, or psychologist treating CeD cannot reassure their patient that gluten contamination does not induce harm to the body. To explain the pathophysiology of celiac disease and the importance of treatment, potential consequences of gluten exposure to patients with CeD (e.g., damage to the intestinal lining) and the benefits of avoidance (e.g., intestinal healing and symptom resolution) are likely to be discussed. This message may be misconstrued by some patients as meaning that dietary hypervigilance is necessary and that symptoms could mean harm.

For some patients, a negative food–symptom association, where symptoms result in hypervigilance, might manifest as follows: Eating a large handful of pistachios creates abdominal discomfort. The postprandial discomfort is interpreted by the brain as the result of likely gluten contamination and, therefore, is perceived as threatening. This thought prompts additional dietary caution to avoid these symptoms again in the future, where the patient may avoid pistachios altogether (e.g., “When I ate pistachios, I experienced symptoms and therefore eating pistachios is not safe.”). In some cases, this interpretation of gluten contamination is correct, and the subsequent restriction may indeed be beneficial. However, this is not always the case, especially if there is no gluten contamination. In the case above, restriction could be falsely attributed to the contamination of the pistachios with gluten, when the real culprit for symptoms was eating an excessive number of pistachios at one time. Incorrectly attributing post-prandial symptoms to gluten and perceiving the situation as dangerous even when it is not could prompt unnecessary food avoidance. This cycle could increase the risk of developing more problematic or generalized food avoidance or restriction, as is seen in ARFID.

Physicians and dietitians should be aware of this potential for negative food–symptom association and the language used when discussing the risks of gluten contamination with patients with CeD, as it may help to prevent the development of ARFID. We suggest asking patients questions about what symptoms they attribute to gluten contamination, how often they find themselves experiencing what they consider to be contamination, and the amount of fear they experience related to managing their CeD, to begin to assess the impact of food-related fear on symptom management. At times, the distinction between maintaining a careful and strict GFD and engaging in maladaptive eating behaviors may be subtle. Therefore, it is imperative that individuals who are started on a GFD be closely monitored. In the future, urine gluten immunogenic peptides detection kits, which can be purchased by patients and used at home, may help distinguish between true and false gluten exposures, helping clinicians to navigate the patients toward a healthy GFD and away from over-restriction and hypervigilance.

It is encouraging to know that, in studies on patients with CeD, there were no differences identified in bone disease, micronutrient deficiencies, or biopsy findings between those with or without ARFID [21]. Patients with CeD and ARFID did not appear to have a greater adherence to GFD compared to those without ARFID [21]. These findings suggest that maladaptive eating behaviors like those seen in ARFID are not necessary for CeD control and good outcomes; this information can be shared by physicians, dietitians, and psychologists with patients. The follow-up period for the study was short—2 years—but this early data is promising in that it shows that extremes in restriction or avoidance do not lead to better clinical outcomes.

## 7. Clinical Features

Given the potential for an increased risk of ARFID in CeD, it is important for clinicians to be able to recognize it, especially given the overlapping symptoms between the two. Adult patients with ARFID may present with symptoms like early satiety, abdominal pain, nausea, vomiting, diarrhea, or weight loss, and a fear of worsening symptoms with food intake. While the physiologic symptoms may be similar, CeD symptoms are provoked by gluten exposure, whereas symptoms of ARFID may present, regardless of food intake, and may present with a higher psychological burden. Patients with ARFID may limit the quantity of food they eat and limit themselves to certain “safe” foods. Additionally, they may be embarrassed or insecure about this behavior and may eat privately to avoid judgment [8]. This may lead to the avoidance of social gatherings, such as parties or dinners, which may eventually lead to social isolation [4]. One study of adult ARFID patients admitted to an in-patient hospital service suggested that the most common clinical presentation of ARFID was the fear of adverse physical consequences of eating. The most common laboratory abnormalities seen in these patients were hypokalemia, vitamin D deficiency, leukopenia, elevated serum bicarbonate, and low serum prealbumin [22].

## 8. Differential Diagnoses

Restrictive eating is a non-specific symptom that may be secondary to various psychiatric conditions or multiple medical conditions. Therefore, it is important to entertain a comprehensive differential diagnosis prior to diagnosing ARFID, depending on the patient’s age, symptoms, and comorbidities (Table 2) [10].

Restrictive eating can be a sign of a psychiatric condition, and this must be considered prior to diagnosing ARFID. Anorexia Nervosa and Bulimia Nervosa may present similarly to ARFID; however, the key distinction is that patients with ARFID do not display a fear of weight gain, persistent behaviors to lose weight, or a disturbed perception of their body weight and shape. Patients with Obsessive–Compulsive Disorder (OCD) may also display restrictive eating habits due to preoccupations with food or ritualized diet patterns. Individuals with autism spectrum disorder may display avoidant food behaviors secondary to heightened sensory activity or rigid eating behaviors. Some patients may also present with food avoidance secondary to anxiety or specific phobias. These may occur secondary to a fear of choking or vomiting, leading to avoiding food intake in general.

There are medical conditions to consider as well—notably, CeD. The potential for overlap between gastrointestinal conditions and ARFID makes the diagnosis particularly challenging. Consider a wide array of possibilities in the evaluation and refer to the *DSM-5* definition seen in Table 1 when making a diagnosis of ARFID.

## 9. Complications

Once the clinical features are recognized and ARFID is diagnosed, individuals with ARFID in CeD may experience several complications as the restrictive eating pattern is extended further than a GFD. Some of these complications involve mental health and some involve physical health. Excessive food avoidance can lead to higher rates of depression and anxiety, decreased social and physical functioning, and higher nutritional and caloric deficiencies [4,8].

Research has shown that individuals with CeD following a GFD often disproportionately experience psychological and social complications, which are often pronounced in individuals exhibiting ARFID traits. A cross-sectional study of 538 patients with CeD in New York City highlights that individuals with CeD adhering to a GFD for a shorter duration tend to report higher levels of anxiety, maladaptive eating behaviors, and reduced quality of life [4]. Social anxiety was prevalent, with nearly 10% meeting the clinical criteria for a social anxiety disorder [4]. Certain demographics, such as younger adults aged 23–35, individuals who are single, and females, were found to be at greater risk of experiencing higher anxiety and poorer eating attitudes. Some patients avoiding additional foods without diagnosed allergies or intolerances had higher rates of depression, with over 75% meeting the clinical threshold for depressive symptoms. These findings underscore the significant emotional burden and social impact that food restriction can impose and reinforce the importance of developing targeted interventions to enhance well-being and promote balanced eating behaviors [23].

Studies investigating ARFID behaviors in patients with digestive diseases, including CeD, further highlight these potential complications and concerns [10]. Patients with ARFID symptoms were found to have heightened levels of anxiety and depression, alongside reduced health-related quality of life. Notably, the strongest correlation was observed between ARFID symptoms and impaired social functioning, indicating that individuals with ARFID are more prone to social withdrawal and may struggle to engage with others. This social isolation may stem from heightened anxiety around food, fear of adverse gastrointestinal symptoms, or difficulty navigating social situations involving food where dietary restrictions are on display [8].

Furthermore, ARFID may exacerbate the medical complications associated with CeD. Micronutrient deficiencies are already common in individuals with CeD, arising from both intestinal malabsorption and the inherently restrictive nature of a GFD. Essential nutrients such as iron, vitamin B12, folate, zinc, and vitamin D are particularly susceptible to depletion [24]. When individuals with CeD further limit their food choices as a result of ARFID, they may have an increased risk of developing these deficiencies [25,26]. These deficiencies can, in turn, contribute to a range of medical complications. Inadequate vitamin D and calcium levels can impair bone mineralization, heightening the risk of osteopenia, osteoporosis, and fractures [27]. Iron deficiency may lead to anemia, resulting in fatigue, weakness, and impaired cognitive function. Folate and vitamin B12 deficiencies can further compromise neurological health, potentially causing peripheral neuropathy, mood disturbances, and cognitive impairment. Zinc deficiency may weaken immune function, increasing susceptibility to infections and delaying wound healing. Zinc deficiency may also result in changes in taste, or ageusia, further altering the individual’s relationship with food. Long-term caloric deficiency during childhood and adolescence may result in delayed puberty and stunted growth [28]. Importantly, exacerbations of nutritional deficiencies in the face of additional health stressors may precipitate acute electrolyte imbalance, dehydration, and even cardiac arrest. Therefore, it is imperative to have a structured approach to monitoring nutrient levels and dietary intake, guided by dietitians, that ensures the proper management of these deficiencies [2,29,30].

## 10. Management

Given the rising prevalence of ARFID and its significant medical complications and psychosocial impacts in both adult and pediatric populations, its early identification and management is necessary. A multidisciplinary team should be assembled to treat patients with ARFID and CeD. Key multimodal approaches include regular monitoring of medical symptoms and complications by primary care physicians and gastroenterologists, education on a healthy GFD, which may include structured meal plans by dietitians, and therapies like Cognitive Behavioral Therapy (CBT) and exposure therapy directed by psychologists [26,31].

Physicians have a role in ARFID and CeD beyond recognizing clinical features and assisting in the diagnosis. They are responsible for regular medical monitoring. Gastroenterologists should closely follow the patients’ adherence to the GFD, symptoms, serological and histological markers of CeD, and overall nutritional status. Detecting and managing micronutrient deficiencies, which are a common complication of CeD, are also in their purview. Checking vitamin levels at CeD diagnosis, recommending supplements, following deficient levels until normalized, and periodically screening for micronutrient deficiencies are the physician’s responsibility. Additionally, patients with CeD complicated by ARFID are at a higher risk of restrictive eating and require closer follow-up with their gastroenterologists, dietitians, and PCPs for monitoring of nutritional intake and potential deficiencies, weight trends, and attitude towards eating habits. For children and adolescents with ARFID and CeD, pediatricians have a crucial role assessing growth parameters, given the risk for malnutrition, weight loss, and impaired growth when these conditions coexist [6]. For those with CeD with or without ARFID, longitudinal care can be shared by the primary care physician (or pediatrician) and gastroenterologist, as close follow-up is imperative.

Awareness of indications for hospitalization when a patient exhibits severe symptoms is important for the treating physician [32,33]. These include dehydration, electrolyte imbalances (hypokalemia, hyponatremia, hypophosphatemia), significant bradycardia (<50 bpm), hypotension (<90/45 mmHg), hypothermia (<96 °F or 35.6 °C), complete food refusal, failed outpatient treatment attempts, and malnutrition-related medical complications such as syncope, seizures, cardiac failure, or pancreatitis. These alarm signs should prompt inpatient management. This environment offers an opportunity for more intense multidisciplinary care and careful monitoring for refeeding [32,33].

Dietary modifications are essential for managing CeD, and dietitians play a key role in guiding patients to select nutrient-dense, gluten-free foods to maintain a balanced diet, prevent micronutrient deficiencies, and preserve food-related quality of life. During the nutrition assessment, dietitians should screen individuals with CeD for the avoidance or restriction of food disproportionate to what would be expected for CeD. By developing personalized nutrition care plans that consider the patient’s health history, dietary preferences, cultural factors, and socioeconomic status, dietitians can help patients maintain a GFD while minimizing the fear associated with eating [2,29,34,35].

**Figure 1:**
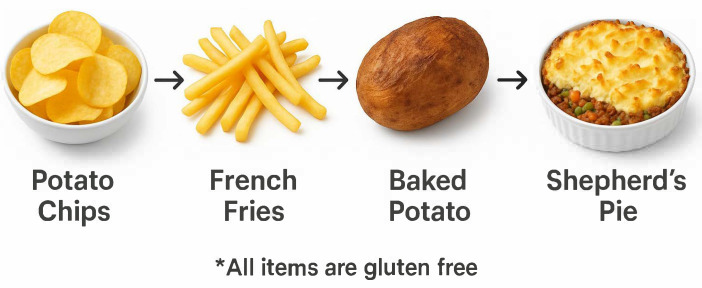
Example of food chaining in ARFID and celiac disease.

One technique, known as food chaining (Figure 1), has recently been discussed for the management of patients with ARFID, especially in children. Food chaining is a systematic method to provide patients with an individualized and safe feeding program that can be implemented at home. The goal of food chaining is to gradually expand the variety of food groups in the patient’s diet by emphasizing the similarities between accepted and targeted foods [36] to reduce anxiety around new food groups that allow for increased exposure to new textures and novel items [36,37].

Dietitians also have a role in an educational capacity for patients with CeD and ARFID. Low dietary literacy has been associated with ARFID symptoms in patients with inflammatory bowel disease [38]. Dietary literacy reflects a patient’s ability to find alternatives when presented with dietary restrictions. This concept is also relevant to CeD. When presented with the need to follow a GFD, patients with low dietary literacy may be overwhelmed and be prone to over-restricting their diet. This worry may lead to limiting to known safe foods, both when preparing food at home or eating in public. Patients may avoid eating in public due to concerns about what food to order and how to order it in a way that feels safe to their bodies and their ego (concerns about how they will be perceived). In this case, referral to a specialized gastrointestinal dietitian may help a patient feel confident in their ability to find and prepare gluten-free alternatives and ultimately liberalize their diet. If significant avoidance behaviors are present, it may be appropriate to refer a patient to a gastrointestinal psychologist, who can help the patient address symptom-specific anxiety and reduce the avoidance behaviors associated with managing dietary restrictions. We encourage physicians treating CeD to inquire about how challenging patients find it to navigate a GFD and to make appropriate referrals to a gastrointestinal dietitian or psychologist when necessary [38].

A psychologist with experience in gastrointestinal disorders is another resource and member of the treatment team for CeD and ARFID patients. Cognitive Behavioral Therapy (CBT) may be useful in patients with ARFID, as it can help target the specific motivations behind restrictive eating habits by modifying dysfunctional cognitions. For example, psychologists may work closely with patients to identify specific fears, then use a structured exposure therapy technique to help patients identify the discrepancy between their expected outcome and actual outcome. Over time, the new association between the feared situation and actual outcome replaces the older association [34,37].

There is limited data on the use of pharmacological techniques for the management of ARFID, especially in patients with CeD. The key management techniques for ARFID should focus on dietary and behavioral therapies, with medical management of any medical complications. Pharmacological therapy may be considered when there is co-existing psychiatric comorbidity, such as anxiety or depression [39].

Close follow-up and a comprehensive approach to address both the physical and psychological aspects of CeD and ARFID is essential for optimizing overall health outcomes [5,6,7,8].

## 11. Conclusions

ARFID represents an underrecognized but clinically significant co-morbid condition in patients with CeD. There are aspects of CeD management with the GFD that may predispose patients to ARFID, and the clinical presentation of the two conditions may overlap. The presence of ARFID can amplify nutritional deficiencies, psychological distress, and social isolation, undermining the benefits of a GFD. Accurate diagnosis requires distinguishing adaptive dietary behavior from pathological restriction. A multidisciplinary approach incorporating medical, dietary, and psychological support is essential for effective management. Further research is needed to develop validated screening tools and targeted interventions specific to this population.

## Figures and Tables

**Table 1:** Diagnostic criteria (adapted from *DSM-5*) for ARFID [9].

Section	Criteria
**A. Core Features**	An eating or feeding disturbance (e.g., apparent lack of interest in eating or food; avoidance based on sensory characteristics of food; concern about aversive consequences of eating) associated with one or more of the following:
	1. Significant weight loss (or failure to achieve expected weight gain/faltering growth in children).
	2. Significant nutritional deficiency.
	3. Dependence on enteral feeding or oral nutritional supplements
	4. Marked interference with psychosocial functioning.
**B. Food Access and Culture**	The disturbance is not better explained by lack of available food or by a culturally sanctioned practice.
**C. Differential Diagnosis**	The disturbance does not occur exclusively during anorexia nervosa or bulimia nervosa, and there is no body image disturbance.
**D. Medical/Psychiatric Attribution**	The disturbance is not attributable to a medical condition or another mental disorder. If it co-occurs with another condition, its severity warrants separate clinical attention.

**Table 2:** Differential diagnosis of ARFID [10].

Differential Diagnosis
**Psychiatric or behavioral conditions:** o Anorexia nervosa o Bulimia nervosa o Autism spectrum disorders (where food selectivity is common) o Anxiety disorders o Major depressive disorder (with changes in appetite or weight) o Social phobia (may affect eating in public settings) o OCD o Post-traumatic stress disorder, following a traumatic event related to eating or food o ADHD, when associated with impulsivity affecting eating behaviors o Rumination disorder
**Gastrointestinal conditions:** o Gastroesophageal reflux disease o Eosinophilic esophagitis o Inflammatory bowel disease o Celiac disease o Chronic idiopathic constipation o Chronic mesenteric ischemia
**Other medical conditions:** o Pica o Food allergies o Neurodevelopmental disorders with sensory sensitivities affecting feeding o Structural anomalies of oropharynx or gastrointestinal tract affecting intake o Endocrine disorder like Addisons disease or hypothyroidism that affect appetite

## Data Availability

Data sharing is not applicable.
